# Supplementary material for: Autoantibodies against chemokines post-SARS-CoV-2 infection correlate with disease course
Source: Nat Immunol. 2023 Mar 6;24(4):604–11. doi: 10.1038/s41590-023-01445-w (PMC10063443; doi:10.1038/s41590-023-01445-w)
Supplement: Supplementary file 1 [file 41590_2023_1445_MOESM1_ESM.pdf]

nature portfolio

Corresponding author(s): Davide F. Robbiani, Mariagrazia Uguccioni, Andrea Cavalli

Last updated by author(s): 24/01/23

Reporting Summary

Nature Portfolio wishes to improve the reproducibility of the work that we publish. This form provides structure for consistency and transparency in reporting. For further information on Nature Portfolio policies, see our Editorial Policies and the Editorial Policy Checklist.

Statistics

For all statistical analyses, confirm that the following items are present in the figure legend, table legend, main text, or Methods section.

n/a	Confirmed	
☐	☒	The exact sample size (*n*) for each experimental group/condition, given as a discrete number and unit of measurement
☐	☒	A statement on whether measurements were taken from distinct samples or whether the same sample was measured repeatedly
☐	☒	The statistical test(s) used AND whether they are one- or two-sided<br>*Only common tests should be described solely by name; describe more complex techniques in the Methods section.*
☒	☐	A description of all covariates tested
☐	☒	A description of any assumptions or corrections, such as tests of normality and adjustment for multiple comparisons
☐	☒	A full description of the statistical parameters including central tendency (e.g. means) or other basic estimates (e.g. regression coefficient) AND variation (e.g. standard deviation) or associated estimates of uncertainty (e.g. confidence intervals)
☐	☒	For null hypothesis testing, the test statistic (e.g. *F*, *t*, *r*) with confidence intervals, effect sizes, degrees of freedom and *P* value noted<br>*Give P values as exact values whenever suitable.*
☒	☐	For Bayesian analysis, information on the choice of priors and Markov chain Monte Carlo settings
☒	☐	For hierarchical and complex designs, identification of the appropriate level for tests and full reporting of outcomes
☐	☒	Estimates of effect sizes (e.g. Cohen's *d*, Pearson's *r*), indicating how they were calculated

Our web collection on statistics for biologists contains articles on many of the points above.

Software and code

Policy information about availability of computer code

Data collection	- Flow cytometry data were collected with the FACS Aria III instrument.<br>- ELISA data were obtained with an ELISA microplate reader (from BioTek) with the Gen5 software.<br>- Nanoluc Luciferase activity in cell lysates was measured using the Nano-Glo Luciferase Assay System (Promega) with Modulus II Microplate Reader User interface (TURNER BioSystems).
Data analysis	For data analysis, the following software were used:<br>- FlowJo Software (version 10.7.1)<br>- Gen5 Software (version 3.12; Agilent)<br>- Glomax software (version 3.2.3; Promega)<br>- IgPipeline (as described in Robbiani et al., 2020)<br>- Prism 9 (version 9.0.2; GraphPad Software)<br>- Pretty Heatmaps (pheatmap) R package v 1.0.12<br>- PyMOL 2.5.0 (Schrödinger, Inc.)<br>- Rtsne R package v 0.15<br>- R 4.1.1 (R Development Core Team)<br>- RStudio 2021.09.0 (RStudio)<br>- Adobe Illustrator 2023<br>- Code availability: https://github.com/cavallilab/chemopept

For manuscripts utilizing custom algorithms or software that are central to the research but not yet described in published literature, software must be made available to editors and reviewers. We strongly encourage code deposition in a community repository (e.g. GitHub). See the Nature Portfolio guidelines for submitting code & software for further information.

Data

Policy information about availability of data

All manuscripts must include a data availability statement. This statement should provide the following information, where applicable:

- Accession codes, unique identifiers, or web links for publicly available datasets
- A description of any restrictions on data availability
- For clinical datasets or third party data, please ensure that the statement adheres to our policy

All data analyzed during the present study are included in this article and its supporting information files. Source data are provided with this paper. PDB accession codes are 5T1A, 6MEO, 6WWZ, and 1ESR.

Human research participants

Policy information about studies involving human research participants and Sex and Gender in Research.

Reporting on sex and gender

Sex/gender was not considered for the study design, which was based on sample availability. Data were however analyzed to determine whether sex/gender were confounders. The main findings described in this study apply to all Sex/genders.

Population characteristics

All the demographics and clinical characteristics of the participants can be found in Supplementary Table 1.

Recruitment

COVID-19 original cohort (Lugano):
71 participants, diagnosed with COVID-19 at the Clinica Luganese Moncucco (CLM, Switzerland) between 08.03.2020 and 22.11.2020, were enrolled in the study and divided into two groups, according to the severity of the acute disease. The hospitalized group included 50 participants; the outpatient group included 21 close contacts of the hospitalized group, who only received at-home care. Inclusion criteria for the hospitalized group were a SARS-CoV-2 positive nasopharyngeal swab test by real-time reverse transcription-polymerase chain reaction (RT PCR) and age ≥18 years. Inclusion criteria for the outpatient group were being a symptomatic close contact (living in the same household) of an individual enrolled in the hospitalized group and age ≥18 years. Serologic tests confirmed COVID-19 positivity for all the participants (Fig. 1a; Extended Data Fig. 4a). At the 12-month visit, participants were asked to indicate the presence or absence of persisting symptoms related to COVID-19 according to a questionnaire. The study was performed in compliance with all relevant ethical regulations and the study protocols were approved by the Ethical Committee of the Canton Ticino (ECCT): CE-3428 and CE 3960.

COVID-19 validation cohort 1 (Milan):
44 participants, diagnosed with COVID-19 and hospitalized at the Humanitas Research Hospital (Milan, Italy) between 10.03.2020 and 29.03.2021, were enrolled in the study. Inclusion criteria for the participants were a SARS-CoV-2 positive nasopharyngeal swab test by RT-PCR and age ≥18 years. Serologic tests confirmed COVID-19 positivity for the participants who were not tested by RT-PCR. The study was performed in compliance with all relevant ethical regulations and the study protocols were approved by the Ethical Committee of Humanitas Research Hospital (authorization n° 738/20 and n° 956/20).

COVID-19 validation cohort 2 (Zurich):
104 participants, diagnosed with COVID-19 at the University Hospital Zurich, the City Hospital Triemli Zurich, the Limmattal Hospital or the Uster Hospital between April 2020 and April 2021, were included in the study and divided into two groups, according to the severity of the acute disease. The hospitalized group included 38 participants, whereas the outpatient group included 66 individuals, who only received at-home care. Inclusion criteria for the participants were a SARS-CoV-2 positive nasopharyngeal swab test by RT-PCR and age ≥18 years. At the 13-month visit, blood was collected, and participants were asked by trained study physicians to indicate the presence or absence of persisting symptoms related to COVID-19. The study was performed in compliance with all relevant ethical regulations and the study protocols were approved by the Cantonal Ethics Committee of Zurich (BASEC #2016-01440).

Control cohort:
15 adult participants (≥18 years) with self-reported absence of prior SARS CoV-2 infection or vaccination (confirmed by negative serologic test, fig. S3A) were enrolled between November 2020 and June 2021. Additional 8 pre-pandemic samples were obtained from blood bank donors (ECCT: CE-3428).

Vaccination cohort:
16 adult participants (≥18 years) with self-reported absence of prior SARS-CoV-2 infection (confirmed by negative serologic test, fig. S4F) and who received two doses of mRNA-based COVID-19 vaccine 62, 63, were enrolled on the day of first vaccine dose or earlier, between November 2020 and October 2021 (ECCT: CE-3428).

HIV-1 and autoimmune diseases cohorts:
Pre-pandemic plasma samples were obtained from the following participants: 24 HIV-1 positive (ECCT: CE-813) 64, 13 each with Ankylosing Spondylitis, Rheumatoid Arthritis (ECCT: CE 3065, and Ethical Committee of the Canton Zurich EK-515), or Sjögren's syndrome (IRCCS Policlinico San Matteo Foundation Ethics Committee n.20070001302).

Lyme disease cohort:
Plasma samples of 27 individuals with erythema migrans (Lyme disease) and 30 controls were obtained at The Valley Hospital (Ridgewood, NJ, USA) and Lyme & Tick-borne Disease Research Center at Columbia University Irving Medical Center (New York, NY, USA) between 2015 and 2019. All were between 18-89 years of age and all denied being immunocompromised. Lyme disease cohort: Individuals had new or recent onset erythema migrans, exposure to a Lyme endemic area in the prior

	30 days and received no more than 3 weeks of antibiotic treatment. Healthy control cohort: Individuals reported being medically healthy, had an unremarkable physical exam and blood tests, had no signs or symptoms of infection or illness, denied having had a diagnosis and/or treatment for Lyme and/or another tick-borne disease within the past 5 years, and denied having a tick bite in the prior 6 months. The Lyme cohort samples were collected at the time of the erythema migrans and 6 months later in average. The study was performed in compliance with all relevant ethical regulations and the study protocol was approved by the New York State Psychiatric Institute Institutional Review Board (#6805).
Ethics oversight	COVID-19 original cohort (Lugano): The study was performed in compliance with all relevant ethical regulations and the study protocols were approved by the Ethical Committee of the Canton Ticino (ECCT): CE-3428 and CE 3960.<br><br>COVID-19 validation cohort 1 (Milan): The study was performed in compliance with all relevant ethical regulations and the study protocols were approved by the Ethical Committee of Humanitas Research Hospital (authorization n° 738/20 and n° 956/20).<br><br>COVID-19 validation cohort 2 (Zurich): The study was performed in compliance with all relevant ethical regulations and the study protocols were approved by the Cantonal Ethics Committee of Zurich (BASEC #2016-01440).<br><br>Control cohort: ECCT: CE-3428.<br><br>Vaccination cohort: ECCT: CE-3428<br><br>HIV-1 and autoimmune diseases cohorts: HIV-1 (ECCT: CE-813); Ankylosing Spondylitis and Rheumatoid Arthritis (ECCT: CE 3065, and Ethical Committee of the Canton Zurich EK-515); and Sjögren's syndrome (IRCCS Policlinico San Matteo Foundation Ethics Committee n.20070001302).<br><br>Lyme disease cohort: The study was performed in compliance with all relevant ethical regulations and the study protocol was approved by the New York State Psychiatric Institute Institutional Review Board (#6805).<br><br>Written informed consent was obtained from all participants, and all samples were coded to remove identifiers at the time of blood withdrawal. Demographic, clinical, and serological features are reported in supplementary tables.

Note that full information on the approval of the study protocol must also be provided in the manuscript.

Field-specific reporting

Please select the one below that is the best fit for your research. If you are not sure, read the appropriate sections before making your selection.

☒ Life sciences ☐ Behavioural & social sciences ☐ Ecological, evolutionary & environmental sciences

For a reference copy of the document with all sections, see nature.com/documents/nr-reporting-summary-flat.pdf

Life sciences study design

All studies must disclose on these points even when the disclosure is negative.

Sample size	No tests were used to determine sample size. Sample size was determined by the number of samples that were available to us and that fit the criteria of the study.
Data exclusions	No data were excluded.
Replication	All results were performed in at least two or three independent experiments as described in each figure legend.
Randomization	Randomization does not apply to this study. Experimental groups were determined by disease status (control vs COVID-19), disease severity (outpatient vs hospitalized COVID-19 individuals), vaccination status, patient clinical outcome (development of Long COVID or not), and disease groups (COVID-19 vs HIV-1 vs autoimmunity).
Blinding	The operator was blind to the assignment of a patient sample to disease group.

Reporting for specific materials, systems and methods

We require information from authors about some types of materials, experimental systems and methods used in many studies. Here, indicate whether each material, system or method listed is relevant to your study. If you are not sure if a list item applies to your research, read the appropriate section before selecting a response.

Materials & experimental systems

n/a	Involved in the study	
☐	☒	Antibodies
☐	☒	Eukaryotic cell lines
☒	☐	Palaeontology and archaeology
☒	☐	Animals and other organisms
☒	☐	Clinical data
☒	☐	Dual use research of concern

Methods

n/a	Involved in the study	
☒	☐	ChIP-seq
☐	☒	Flow cytometry
☒	☐	MRI-based neuroimaging

Antibodies

Antibodies used

Commercially available antibodies:
- Anti-human CD14, APC-eFluor780, clone 61D3 (Thermo Fisher Scientific; Cat#47-0149-42; RRID:AB_1834358; dilution 1:200)
- Anti-human CD16, APC-eFluor780, clone eBioCB16 (CB16; Thermo Fisher Scientific; Cat#47-0168-41; RRID:AB_11219083; dilution 1:200)
- Anti-human CD20, PE-Cy7, clone L27 (IVD; BD Biosciences; Cat#335828; RRID:AB_2868689; dilution 1:200)
- Anti-human CD3, APC-eFluor780, clone OKT3 (Thermo Fisher Scientific; Cat#47-0037-41; RRID:AB_2573935; dilution 1:200)
- Anti-human CD8a, APC-eFluor780, clone OKT8 (Thermo Fisher Scientific; Cat#47-0086-42; RRID:AB_2573945; dilution 1:200)
- Anti-human IgG, HRP-linked whole Ab (GE Healthcare; Cat# NA933; RRID:AB_772208; dilution 1:5000)

Monoclonal antibodies produced in this study: aCCL8.001, aCCL8.003, aCCL8.004, aCCL8.005, aCCL20.001, aCXCL13.001, aCXCL13.002, aCXCL13.003, aCXCL16.001, aCXCL16.002 and aCXCL16.003

Isotype control used in this study: Z021 (see Robbiani et al., 2017 as described in the method section).

Validation

All the commercially available antibodies utilized in this study have been validated by the manufacturer. Details can be found on their websites:
- Anti-human CD14, APC-eFluor780, clone 61D3 (Thermo Fisher Scientific; Cat#47-0149-42; RRID:AB_1834358; dilution 1:200)
  Website: https://www.thermofisher.com/antibody/product/CD14-Antibody-clone-61D3-Monoclonal/47-0149-42
- Anti-human CD16, APC-eFluor780, clone eBioCB16 (CB16; Thermo Fisher Scientific; Cat#47-0168-41; RRID:AB_11219083; dilution 1:200)
  Website: https://www.thermofisher.com/antibody/product/CD16-Antibody-clone-eBioCB16-CB16-Monoclonal/47-0168-41
- Anti-human CD20, PE-Cy7, clone L27 (IVD; BD Biosciences; Cat#335828; RRID:AB_2868689; dilution 1:200)
  Website: https://www.bdbiosciences.com/en-ch/products/reagents/flow-cytometry-reagents/clinical-diagnostics/single-color-antibodies-asr-ivd-ce-ivd/cd20-pe-cy-7.335828
- Anti-human CD3, APC-eFluor780, clone OKT3 (Thermo Fisher Scientific; Cat#47-0037-41; RRID:AB_2573935; dilution 1:200)
  Website: https://www.thermofisher.com/antibody/product/CD3-Antibody-clone-OKT3-Monoclonal/47-0037-41
- Anti-human CD8a, APC-eFluor780, clone OKT8 (Thermo Fisher Scientific; Cat#47-0086-42; RRID:AB_2573945; dilution 1:200)
  Website: https://www.thermofisher.com/antibody/product/CD8a-Antibody-clone-OKT8-OKT-8-Monoclonal/47-0086-42
- Anti-human IgG, HRP-linked whole Ab (GE Healthcare; Cat# NA933; RRID:AB_772208; dilution 1:5000)
  Website: https://www.fishersci.se/shop/products/anti-human-igg-peroxidase-linked-species-specific-whole-antibody-from-sheep-secondary-antibody-ge-healthcare/10547065

The specificity of the monoclonal antibodies produced in this study has been validated by testing binding to their cognate human antigen in ELISA (Fig. 2d and 2f; Extended Data Fig. 8f and 8i) and by assessing the inhibition of cellular migration toward human chemokines (2e and 2g; Extended Data Fig. 8g and 8j) compared to an isotype control.

Eukaryotic cell lines

Policy information about cell lines and Sex and Gender in Research

Cell line source(s)

- 293T (ACE2)
- HEK293T (ATCC CRL-11268)
- Expi293F
- PreB 300.19 murine cell line expressing hCCR2 (Ogilvie at al, 2001)
- PreB 300.19 murine cell line expressing hCCR6 (Ogilvie at al, 2001)
- PreB 300.19 murine cell line expressing hCXCR1 (Zaslaver at al, 2001)
- PreB 300.19 murine cell line expressing hCXCR6 (Loetscher at al, 1997)

Authentication

No authentication was performed for the commercially available cell lines.
Expression of the cognate chemokine receptor on PreB 300.19 murine cell lines was confirmed by flow cytometry, and selective activity of the human agonist assessed by in vitro migration.

Mycoplasma contamination

Cell lines were not tested for Mycoplasma.

Commonly misidentified lines
(See ICLAC register)

No commonly misidentified cell lines were used.

Flow Cytometry

Plots

Confirm that:

☒ The axis labels state the marker and fluorochrome used (e.g. CD4-FITC).

☒ The axis scales are clearly visible. Include numbers along axes only for bottom left plot of group (a 'group' is an analysis of identical markers).

☒ All plots are contour plots with outliers or pseudocolor plots.

☒ A numerical value for number of cells or percentage (with statistics) is provided.

Methodology

Sample preparation	B cells were enriched from PBMCs of uninfected controls or of COVID-19 convalescent individuals 6 months after COVID-19, using the pan-B-cell isolation kit according to manufacturer's instructions (Miltenyi Biotec, 130-101-638). The enriched B cells were subsequently stained in FACS buffer (PBS + 2% FCS + 1mM EDTA) with the following antibodies/reagents (all 1:200 diluted) for 30 min on ice: anti-CD20-PE-Cy7 (BD Biosciences, 335828), anti-CD14-APC-eFluor 780 (Thermo Fischer Scientific, 47-0149-42), anti-CD16-APC-eFluor 780 (Thermo Fischer Scientific, 47-0168-41), anti-CD3-APC-eFluor 780 (Thermo Fischer Scientific, 47-0037-41), anti-CD8-APC-eFluor 780 (Invitrogen, 47-0086-42), Zombie NIR (BioLegend, 423105), as well as fluorophore-labeled ovalbumin (Ova) and N-loop peptides. Live single Zombie-NIR−CD14−CD16−CD3−CD8−CD20+Ova−N-loop-PE+N-loop-AF647+ B cells were single-cell sorted into 96-well plates containing 4 µl of lysis buffer (0.5× PBS, 10 mM DTT, 3,000 units/ml RNasin Ribonuclease Inhibitors [Promega, N2615]) per well using a FACS Aria III, and the analysis was performed with FlowJo software. The sorted cells were frozen on dry ice and stored at −80 °C.
Instrument	Cell sorting was performed with the FACS Aria III instrument.
Software	Data analysis was performed using the FlowJo Software (version 10.7.1; Three Star).
Cell population abundance	The percentage of each cell population shown in this study is depicted in each the FACS plot.
Gating strategy	Gating strategy is provided in the Supplementary Information. Briefly, all cells were gated on FSC-A/SSC-A, and doublets were eliminated using SSC-W/SSC-A and FSC-H/FSC-A gates. For live/dead discrimination, the Zombie NIR™ Fixable Viability Kit (BioLegend; Cat#423105) was utilized according to the manufacturer's instructions. B cells specific to a particular chemokine peptide were gated as CD14−CD16−CD3−CD8−CD20+Ova−N-loop-PE+N-loop-AF647+.

☒ Tick this box to confirm that a figure exemplifying the gating strategy is provided in the Supplementary Information.
